# Epithelial Cell Migration and Proliferation Patterns During Initial Wound Closure in Normal Mice and an Experimental Model of Limbal Stem Cell Deficiency

**DOI:** 10.1167/iovs.61.10.27

**Published:** 2020-08-13

**Authors:** Sudan Puri, Mingxia Sun, Kazadi N. Mutoji, Tarsis F. Gesteira, Vivien J. Coulson-Thomas

**Affiliations:** College of Optometry, University of Houston, Houston, Texas, United States

**Keywords:** hyaluronan, limbal stem cells, corneal injury, wound healing, epithelial migration, proliferation

## Abstract

**Purpose:**

Establishing the dynamics of corneal wound healing is of vital importance to better understand corneal inflammation, pathology, and corneal regeneration. Numerous studies have made great strides in investigating multiple aspects of corneal wound healing; however, some aspects remain to be elucidated. This study worked toward establishing (1) if epithelial limbal stem cells (LSCs) are necessary for healing all corneal wounds, (2) the mechanism by which epithelial cells migrate toward the wound, and (3) if centrifugal epithelial cell movement exists.

**Methods:**

To establish different aspects of corneal epithelial wound healing we subjected mice lacking hyaluronan synthase 2 (previously shown to lack LSCs) and wild-type mice to different corneal debridement injury models.

**Results:**

Our data show that both LSCs and corneal epithelial cells contribute toward closure of corneal wounds. In wild-type mice, removal of the limbal rim delayed closure of 1.5-mm wounds, and not of 0.75-mm wounds, indicating that smaller wounds do not rely on LSCs as do larger wounds. In mice shown to lack LSCs, removal of the limbal rim did not affect wound healing, irrespective of the wound size. Finally, transient amplifying cells and central epithelial cells move toward a central corneal wound in a centripetal manner, whereas central epithelial cells may move in a centrifugal manner to resurface peripheral corneal wounds.

**Conclusions:**

Our findings show the dimensions of the corneal wound dictate involvement of LSCs. Our data suggest that divergent findings by different groups on the dynamics of wound healing can be in part owing to differences in the wounding models used.

The corneal epithelium is the outermost layer of the cornea and, as such, is exposed to the external environment being prone to scratches and injuries. Defects in the ability to repair and restore epithelial integrity after injury can lead to a loss of corneal transparency and visual impairment. The corneal epithelium constitutes the main barrier for preventing pathogens from entering the corneal stroma and, after wounding, the healing process must occur in an efficient manner to avoid infection that can ultimately compromise corneal transparency. Corneas contain limbal epithelial stem cells (LSCs) that generate new epithelial cells during homeostasis and after wounding, and, are therefore vital for maintaining a healthy corneal epithelium. A decrease or loss of LSCs leads to LSC deficiency (LSCD), which is characterized by a decreased ability to repopulate the corneal epithelium after injury. LSCD is a serious medical condition, with clinical manifestations ranging from corneal opacification, inflammation, vascularization, and severe pain to the complete loss of vision. LSCD is normally diagnosed after the onset of these clinical manifestations owing to misdiagnosis in the early stages. Thus, furthering our understanding of LSCs and how they contribute toward wound closure and, more important, how corneas lacking LSCs heal, is of vital importance.

In the human cornea, LSCs are believed to be located within the basal layer of crypts, the palisades of Vogt, in the limbal region.^1^^–^^6^ These crypts are not present in the mouse cornea^7^; however, numerous studies have elegantly demonstrated that LSCs are located in the basal layer within the limbal region.^8^^–^^12^ LSCs divide to produce progenitor cells, called transit amplifying cells, which proliferate and migrate centripetally toward the central cornea as they differentiate into corneal epithelial cells. Although this is the most accepted paradigm, some studies have suggested that stem cells or oligopotent cells also exist within the peripheral and central cornea.^7^^,^^13^ The understanding of how LSCs contribute to the maintenance of the corneal epithelium remains to be fully established and is of vital importance for understanding the pathogenesis and treatment of LSCD. A plethora of studies have worked toward establishing the exact mechanism by which corneal epithelial wounds heal, but controversy remains around the exact corneal wound healing process. Although most studies show that the healing of corneal epithelial wounds begins with the migration of epithelial cells at the wound edge to resurface the wounded area, there is disagreement on the precise mechanism. A school of thought believes that epithelial cells around the wound margin move in a “sliding”^14^^,^^15^ or “leap frog” motion,^16^ whereas others have indicated that cells at the leading edge of the wound are replaced during epithelial wound healing.^17^ However, most studies agree that there is an increase in epithelial cell proliferation away from the wound edge and in the limbal region that increases the local “density” of cells to “push” epithelial cells along further contributing toward closure of the wound.^14^^,^^16^^,^^18^ Recently, the basal cell migration theory proposes that corneal wounds initially heal by increased population pressure gradient from the limbus to the wound edge that somehow leads to basal epithelial cells moving into the wound bed.^19^

We herein investigated if central corneal wound healing relies exclusively on LSCs, which proliferate and push the epithelial sheet to resurface the corneal epithelium, or if corneal epithelial cells around the wound edge contribute toward wound closure. We also investigated whether injury size dictates the involvement of LSCs in resurfacing the corneal epithelium. We previously showed that the glycosaminoglycan hyaluronan (HA) is necessary for maintaining LSCs in the stem cell phenotype.^20^ Moreover, the knock-out of HA synthase 2 (*Has2*), one of the isoforms of the enzymes responsible for synthesizing HA, was previously shown to lead to the loss of LSCs in mice.^20^ These mice, which have a loss of LSCs, were also used in this study to investigate the role of LSCs in corneal wound healing and how wound healing occurs in this proposed mouse model of LSCD.

## Methods

### Animal Maintenance

C57BL/6J mice, and transgenic mouse lines *K14-rtTA* (K14) (stock number 008099) and *tetO-cre* (TC) (stock number 006224) were obtained from The Jackson Laboratory (Bar Harbor, ME). *K14-rtTA* and *tetO-cre* mice were bred with *Has2* floxed mice (*Has2^flox/flox^*) to generate compound *K14-rtTA*, *tetO-cre* and *tetO-cre Has2^flox/flox^* transgenic mice as previously shown.^20^^–^^22^ The administration of doxycycline chow (Custom Animal Diets, LLC, Easton, PA; 200 mg/kg) was used to induce K14-driven persistent and irreversible excision of *Has2* in triple-transgenic mice *K14-rtTA;TC;Has2^flox/flox^*, hereafter referred to as *Has2^Δ/ΔCorEpi^* mice, which thereby lack *Has2* expression in K14 expressing cells (which include corneal epithelial and limbal epithelial cells), but present *Has2* expression in all other corneal compartments. The identification of each transgenic allele was determined by PCR genotyping with tail DNA using specific primer pairs and all mice in our colony were genotyped. All mice were bred and housed in a temperature-controlled facility with an automatic 12-hour light–dark cycle at the Animal Facility of the University of Houston. Experimental procedures for handling the mice were previously approved by the Institutional Animal Care and Use Committee at the University of Houston. Animal care and use conformed to the ARVO Statement for the Use of Animals in Ophthalmic and Vision Research.

### Circle and Ring Injury Model

**Figure 1.**
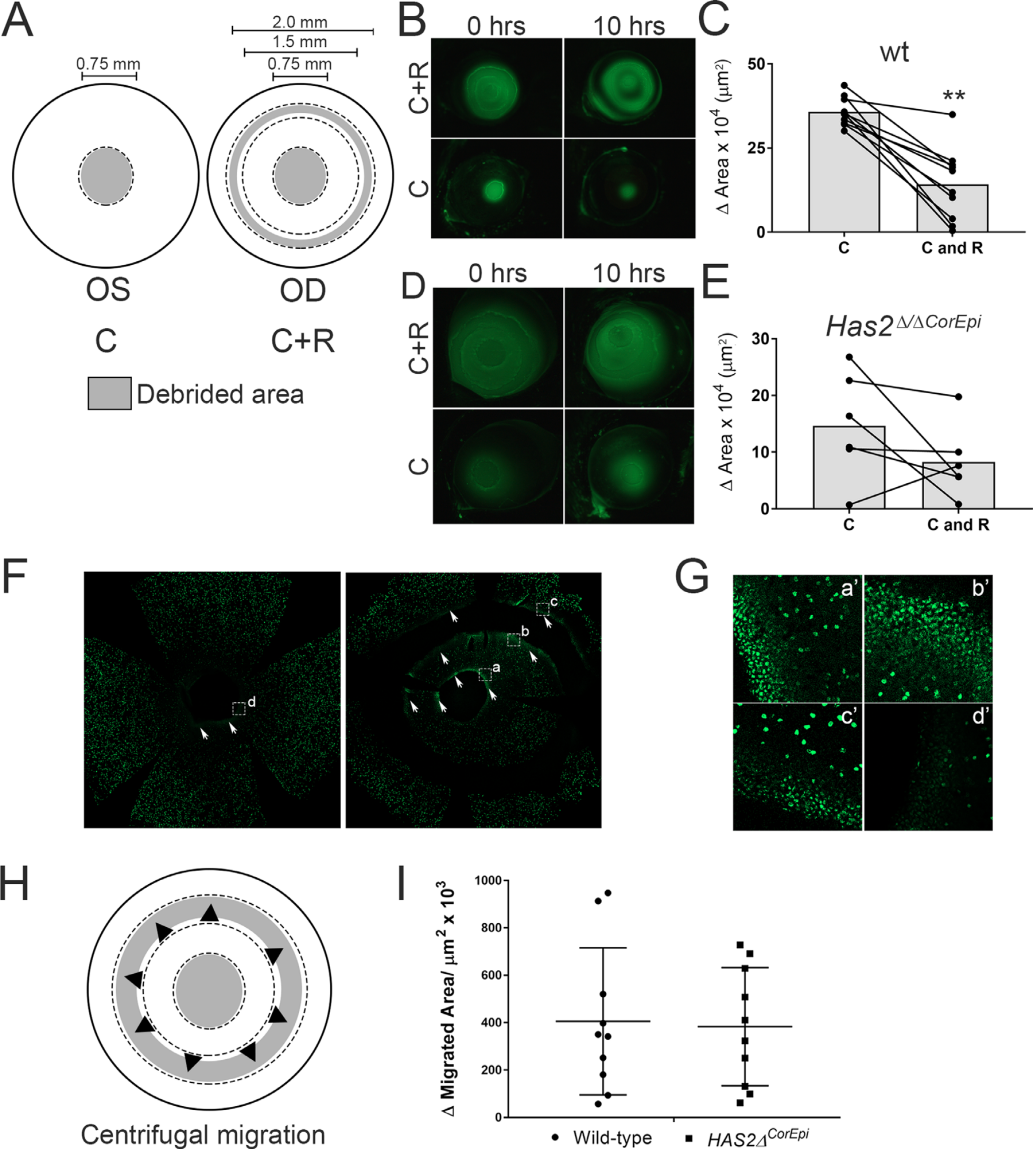
The effect of a peripheral ring wound on healing a central corneal wound in wild-type and *Has2^Δ^^/^^Δ^^CorEpi^* mice. (A) Schematic of the wounding model; the left eye was subjected to a central circular wound of 0.75 mm in diameter, while the right eye was subjected to the same central wound, as well as a ring wound in the peripheral cornea (C+R). The debrided area is represented in gray and spared epithelium in white. (B) Images were acquired of the ocular surface of wild-type mice treated with fluorescein under a stereomicroscope using the GFP filter immediately and 10 hours after injury. (C) The wounded area of wild-type mice was measured immediately and 10 hours after injury and the healed area calculated. Central wounds surrounded by a peripheral ring wound (C+R) presented reduced healed area when compared with corneas with only a circular wound (C). (D) Images were acquired of the ocular surface of *Has2^Δ^^/^^Δ^^CorEpi^* mice treated with fluorescein under a stereomicroscope using the GFP filter immediately and 10 hours after injury. (E) The wounded area was measured immediately and 10 hours after injury and the healed area calculated. The ring wound surrounding the central circular wound (C+R) did not significantly delay wound healing. (F) Images were acquired of EdU (green) stained whole mounted corneas, demarcated areas are shown in higher magnification in (G), corneas were counter stained with DAPI. (H) Schematic representing the centrifugal movement of epithelial cells that was assayed. (I) The migrated area of the wound edge of the innermost rim of the ring wound was calculated for wild-type and *Has2^Δ^^/^^Δ^^CorEpi^* mice to verify whether the ring wound also healed in the central to limbal direction, indicating epithelial cells within the central area are capable of moving in a centrifugal manner. Both wild-type and *Has2^Δ^^/^^Δ^^CorEpi^* mice presented centrifugal epithelial cell movement. No statistically significant difference was found in the centrifugal migrated area between wild-type and *Has2^Δ^^/^^Δ^^CorEpi^* mice. Fluorescein was used to visualize the wounded area and also for the investigator to ascertain the successful removal of cells within the area demarcated with a trephine. Because fluorescein may diffuse throughout the cornea while verifying the quality of the injury, the injured area was measured from the wound edges. ^*^P ≤ 0.05 and ^**^P ≤ 0.01.

Eight- to 10-week-old mice were provided with carprofen gel packs (MediGel CPF – ClearH2O) 24 hours before the procedures and anesthetized by intraperitoneal injection of ketamine hydrochloride (80 mg/kg) and xylazine (10 mg/kg). The eyes were then rinsed with sterile PBS and anesthetized by topical application of 0.5% Proparacaine (Bausch & Lomb, Bridgewater, NJ) to the ocular surface. All injuries were performed at the same time of day to avoid the influence of diurnal changes. Trephines of 0.75 mm, 1.5 mm, and 2.0 mm in diameter (Robbbins Instruments, Chatham, NJ) were concentrically used to demarcate the margins of the epithelial injuries. The epithelium was subsequently removed sparing the basement membrane using an Algerbrush II with a 0.5 mm rotating burr. For the circle and ring injury model (right eye) the epithelium was removed within the 0.75 mm demarcated area and also within the area between the 1.5 mm and 2.0 mm demarcated areas, thereby producing a circular wound within a ring wound (Fig. 1A; the wounded area is in gray). With this injury model, there is an area of intact epithelium between the circular and ring wounds (represented in white). The healing of this injury model was compared with the left eye, which was subjected solely to the central circular wound demarcated with the 0.75 mm trephine. After epithelial debridement, fluorescein solution was to visualize the injured area of the ocular surface and the ocular surface was imaged using the GFP filter under a ZEISS SteREO Discovery. V12 Modular Stereo Microscope (Carl Zeiss Microscopy LLC, Oberkochen, Germany). At 6 hours after injury, the mice were injected with 20 mg/kg 5-ethynyl-2’-deoxyuridine (EdU) intraperitoneally to label proliferating cells. Corneas were reimaged at 10 hours after injury using a fluorescein solution to quantify the wounded area. Eyeballs were enucleated at 10 hours for analysis of EdU-positive (EdU^+^) cells or 48 hours for histologic analysis of corneal stratification.

### Limbus and Central Injury Model

Mice at 8 to 10 weeks of age were provided with carprofen gel packs (MediGel CPF – ClearH_2_O) 24 hours before the procedures and anesthetized by intraperitoneal injection of ketamine hydrochloride (80 mg/kg) and xylazine (10 mg/kg). The eyes were then rinsed with sterile PBS and anesthetized by topical application of 0.5% proparacaine (Bausch & Lomb) to the ocular surface. All injuries were performed at the same time of day to avoid the influence of diurnal changes. The limbal region was removed from the right eye using an Algerbrush II with a 0.5 mm rotating burr sparing the basement membrane while this area was left intact in the left eye, to compare the ability of the cornea to resurface the corneal epithelium with or without LSCs. Thereafter, a trephine of 0.75 mm (small) or 1.5 mm (large) in diameter (Robbbins Instruments) was used to demarcate the margins of the central epithelial injury. The epithelium within the demarcated area was removed sparing the basement membrane using an Algerbrush II with a 0.5 mm rotating burr. Thus, with these injury models, we compared the ability of the cornea to heal large and small wounds with and without LSCs (Fig. 4). After epithelial debridement, 0.1% fluorescein solution was used to ensure that all epithelial cells had been removed within the demarcated area and also to mark the edge of the injured area, and the corneas were imaged using a stereomicroscope with a GFP filter. At 6 hours after injury, the mice were injected with 20 mg/kg EdU intraperitoneally to label proliferating cells. The corneas were imaged again at 10 hours after injury using a fluorescein solution to quantify the change in size of the central injury. Eyeballs were enucleated at 10 hours for analysis of EdU^+^ cells or 48 hours for histologic analysis of corneal stratification.

### Quantification of the Healed Area

Wound healing was estimated by assessing the healed area or migrated distance immediately after injury and at 10 hours after injury. Importantly, we initially assessed the rate of wound healing every 4 hours during a 24-hour period; however, anesthetizing the mouse to capture the image under the stereomicroscope significantly delayed the rate of wound healing; therefore. we opted to select only 1 time-point to measure the healed area/migrated distance. The healed area and migrated distance were calculated from the wound edges and fluorescein staining was used as an indication of epithelial damage. The images were quantified using open-source ImageJ software Fiji 1.52p (National Institutes of Health, Bethesda, MD).^23^ The migrated distance was also calculated by two independent investigators in a blinded manner. The healed area and migrated distance calculated by automated software and by the independent investigators, respectively, yielded corroborating data in all experiments.

### Whole Mount Analysis of Proliferating Cells

Mice were culled 10 hours after injury and eyeballs immediately enucleated and fixed in 4% paraformaldehyde for 30 minutes. Thereafter, eyeballs were washed three times with PBS and corneal buttons excised and four small peripheral incisions made to enable flat mount. Corneas were then treated with the Click-iT EdU labeling kit (Life Technologies Corp., Eugene, OR) according to the manufacturer's recommendation. The corneas were then washed in PBS containing 3% BSA and finally incubated with DAPI (Life Technologies Corporation, Eugene, Oregon) and washed before mounting in Fluoromount-G mounting medium (Southern Biotech, Birmingham, AL). Entire corneas were scanned under a Zeiss LSM 800 confocal microscope with the tiling mode using ZEN 2.3 (blue edition) Imaging software (Carl Zeiss Microscopy LLC). Multiple z-stack tiles were captured encompassing the entire cornea using either the 10× or 20× objective and frames processed together (using the stitching mode followed by full orthogonal projection) into a single image. The number of EdU positive cells (proliferating cells) was manually counted in a blinded manner using Fiji ImageJ software (National Institutes of Health).^23^ For such, the cornea was mapped with concentric circles placed at 0.25-mm intervals and data presented as a percentage of the number of EdU^+^ cells in relation to the total number of cells (DAPI^+^ cells). In Figure 3A, the inner three circles were considered to be the central cornea, the next three circles the peripheral cornea and the outermost circle the limbal region (Supplementary Fig. 2). The number of EdU^+^ cells were counted using automated software generating corroborating data and also by an investigator in a blinded manner. The number of DAPI positive cells was counted using a custom written macro using ImageJ software (Fiji 1.52p, National Institutes of Health).

### Histologic Analysis

Eyeballs were obtained at 10 and 48 hours after injury and processed for histology. The enucleated eyeballs were fixed for 30 minutes in 4% buffered paraformaldehyde, washed with PBS, sequentially dehydrated, immersed in paraffin overnight, and subsequently embedded in blocks. The blocks were sectioned at 5 µm and washed with xylene to remove excess paraffin and then rehydrated. Subsequently, the sections were stained with hematoxylin and eosin. The sections were washed, dehydrated, and mounted in Permount (ThermoFisher Scientific, Waltham, MA). Images were captured using a Leica microscope. For immunohistochemistry, slides containing paraffin sections were placed horizontally and heated at 65°C for 30 minutes, washed three times with xylene to remove excess paraffin and subsequently rehydrated. Cells were permeabilized with 0.01% saponin and nonspecific protein binding sites were blocked with 5% FBS prepared in PBS. Sections were then incubated with the primary antibodies rabbit anti-Krt14 (PRB-155P; Covance, Princeton, NJ), rabbit anti-Krt12 (ab185627; Abcam, Cambridge, MA), rabbit anti-ΔNp63 (ab166857; Abcam), and rabbit anti-Krt8 (ab59400076; Abcam). Sections were then washed in PBS and incubated with secondary antibodies raised in donkey conjugated with Alexa Fluor 488 or Alexa Fluor 555. For HA staining, corneas were incubated with biotinylated HA binding protein (HABP-385911; Millipore, Billerica, MA) followed by NeutrAvidin Alexa 555 (Life Technologies, Carlsbad, CA). Phalloidin Alexa Fluor 647 (Invitrogen) was used to visualize F-actin to identify wound edges. The sections were then washed and the nuclei stained with 40,6-diamidino-2-phenylindole (DAPI; Sigma-Aldrich). Sections were mounted in Fluoromount-G (Electron Microscopy Sciences, Hatfield, PA), imaged using a ZEISS LSM 800 Confocal microscope with Airyscan and analyzed using the Zen Image software (Zeiss).

### Statistics

All experiments were carried out at least three times with at least five mice each time. In the graphs, the lines that connect the dots in each experimental group represent each individual mouse. Both male and female mice were used per experimental group to minimize bias, and data were analyzed separately and together. Image quantification and analysis were performed masked to avoid bias. Differences were assessed by *t* test or ANOVA, followed by post hoc test for multiple comparisons considering *P* < 0.05 as statistically significant.

## Results

### LSCs and Corneal Epithelial Cells Contribute Toward Closure of Central Corneal Wounds

LSCs are believed to be necessary for successful corneal wound healing and it has been proposed that after wounding LSCs proliferate within the limbal region and push the epithelial cell sheet toward the central epithelium thereby resurfacing the injured area.^14^^,^^16^^,^^18^^,^^24^ To evaluate whether LSCs are required during corneal wound healing of a central epithelial wound and also validate whether LSCs push the epithelial cell sheet along to resurface the wounded area we compared two types of injuries. The first injury consisted of a circular central injury 0.75 mm in diameter and the second injury consisted of this same 0.75 mm central wound surrounded by a ring wound in the peripheral region (Fig. 1A). The premise of these injuries is that the ring wound would prevent the epithelial cell sheet from being pushed from the limbal region toward the central cornea, thereby cutting off LSCs as a source of cells for resurfacing the corneal central wound. The ring wound significantly decreased wound healing of the central circular wound from a healed area of approximately 35 × 10^4^ µm^2^ to approximately 15 × 10^4^ µm^2^ in wild-type mice (Figs. 1B and C). However, interestingly, the central wound was still able to heal with the ring wound surrounding it, showing that central and inner peripheral corneal epithelial cells also contribute toward wound closure and, more important, are able to heal the central wound without LSC participation. It is important to note that, with our injury model, we removed a significant portion of peripheral cells, making it difficult to distinguish between the contribution of outer peripheral corneal epithelial cells and LSCs. Therefore, we can infer that LSCs and/or outer corneal peripheral epithelial cells are an important source of cells for resurfacing the epithelium of central corneal wounds; however, they are not required for resurfacing small central corneal wounds. Central corneal epithelial cells surrounding the injury site also contribute toward wound closure of central corneal wounds.

### The Rate of Wound Closure in Has2^Δ/ΔCorEpi^ Mice After Circle and Ring Corneal Wounds

**Figure 2.**
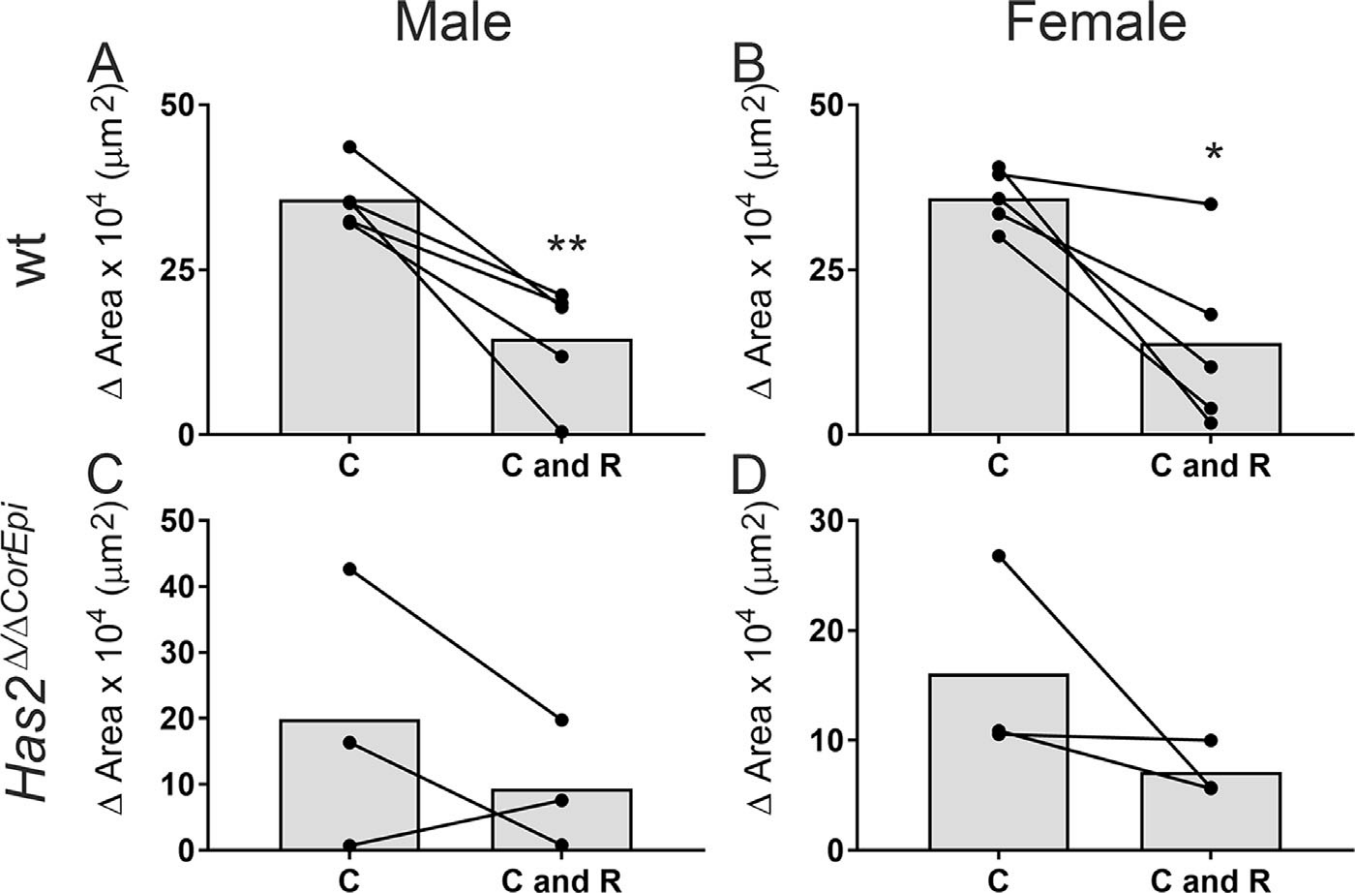
Comparison of the healed area of central wounds surrounded or not surrounded by peripheral ring wounds in *Has2^Δ^^/^^Δ^^CorEpi^* and wild-type mice by gender. The healed area was compared for central wounds surrounded or not surrounded by a peripheral ring wound (C and C+R, respectively) with gender as a variable. (A) Male wild-type mice, (B) female wild-type mice, (C) male *Has2^Δ^^/^^Δ^^CorEpi^* mice, and (D) female *Has2^Δ^^/^^Δ^^CorEpi^* mice all presented reduced wound healing in C+R wounds; however, this difference only achieved significance in the wild-type mice. *P ≤ 0.05 and **P ≤ 0.01.

*Has2^Δ^^/^^Δ^^CorEpi^* mice have been shown to present a loss of LSCs, and thus we have proposed these mice as an experimental mouse model for LSCD.^20^ In this study, we further confirm that *Has2^Δ^^/^^Δ^^CorEpi^* mice, when induced from E0, present a significant loss of LSCs in the limbal region (Supplemental Fig. 1). Specifically, K15-, ΔNp63-, and CK8-positive cells can be seen solely in wild-type mice, whereas *Has2^Δ^^/^^Δ^^CorEpi^* mice present exclusively K12 positive cells in the limbal region (Supplemental Fig. 1).^20^ Moreover, in -wild-type mice CK8-positive cells can be seen within an HA-rich matrix that surrounds limbal epithelial cells; however, this HA-rich matrix is absent in *Has2^Δ^^/^^Δ^^CorEpi^* mice (Supplemental Fig. 1). In *Has2^Δ^^/^^Δ^^CorEpi^* mice, the ring wound did not significantly affect wound healing of central circular wounds. More specifically, the central circular wound (C) presented a healed area of approximately 15 × 10^4^ µm^2^ at 10 hours after injury, whereas a circular wound surrounded by a ring wound (C+R) presented a healed area of approximately 9 × 10^4^ µm^2^ at 10 hours after injury, and this difference did not reach statistical significance (Figs. 2D and E). Thus, *Has2^Δ^^/^^Δ^^CorEpi^* mice that present a loss of LSCs do not rely on cells from the limbal and/or outer peripheral region for healing central circular wounds. Interestingly, when analyzing EdU^+^ cells, it could be noted that some mice presented a high density of proliferating cells at the wound edge, clearly demonstrating that central epithelial cells can proliferate to help resurface the wounded area of central wounds in *Has2^Δ^^/^^Δ^^CorEpi^* mice (Figs. 1F and G).

### Corneal Epithelial Cells Are Capable of Centrifugal Movement

The wound closure of ring wounds revealed that ring wounds are healed from both the limbal and central wound edges in both wild-type and *Has2^Δ^^/^^Δ^^CorEpi^* mice (Figs. 1H and I). Therefore, LSCs and corneal peripheral epithelial cells move in a centripetal manner, from the limbal region toward the central cornea, and central corneal epithelial cells in a centrifugal manner, from the central cornea toward the limbal region. The area healed in a centrifugal manner was calculated for both wild-type and *Has2^Δ^^/^^Δ^^CorEpi^* mice, revealing no significant difference in the rate of centrifugal wound healing (Fig. 1I). Thus, the rate of centrifugal wound healing is not affected in mice with deficient LSCs, whereas the rate of centripetal wound healing is affected in these mice.

### Effect of Gender on Wound Healing Using the Circle and Ring Injury Model

The data obtained in the experiments were also analyzed with gender as a variable (Fig. 2). Similar mean wound healing rates were found for both male and female wild-type mice, and, therefore no differences were noted in terms of gender (Fig. 2). Specifically, in the case of central wounds, both male and female mice showed a healed area of approximately 35 µm^2^, and in the case of central and ring wounds a healed area of approximately 14 µm^2^. Male *Has2^Δ^^/^^Δ^^CorEpi^* mice displayed slightly higher wound healing rates for both C and C+R wounds when compared with female mice (approximately 20 µm^2^ vs. approximately 16 µm^2^ and approximately 10 µm^2^ vs. approximately 7.5 µm^2^, respectively); however, this difference was not statistically significant (Fig. 2).

### The Effect of Wounding on Cell Proliferation in the Circle and Ring Injury Model

**Figure 3.**
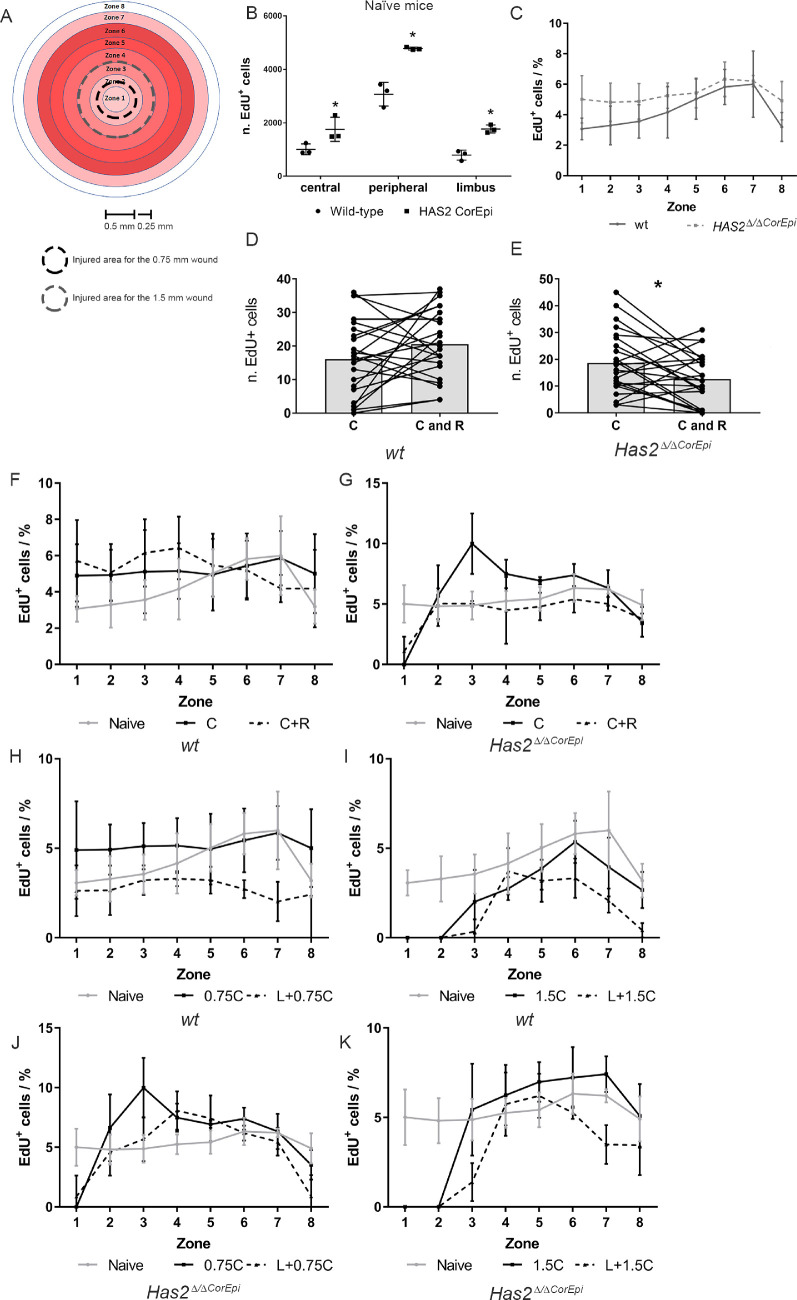
Cell proliferation of naïve and injured corneas after different corneal epithelial wounds. Wild-type and *Has2^Δ^^/^^Δ^^CorEpi^* mice were subjected to different central wounds with or without a ring wound or the concomitant removal of the limbal rim and thereafter labelled with EdU for 4 hours (from 6 to 10 hours after injury). The corneas were obtained 10 hours after injury, processed for whole mount analysis and stained using Click-it EdU Alexa488. The number of EdU^+^ cells was quantified in different zones on the cornea. (A) Schematic representing the different zones of the cornea within which the EdU^+^ cells (proliferating cells) were counted. The injured area for the 0.75 mm and 1.5 mm wounds are demarcated (black dashed line and gray dashed line, respectively). The EdU^+^ cells were counted in the different zones of wild-type and *Has2^Δ^^/^^Δ^^CorEpi^* mice different corneal wounds. (B) The number of EdU^+^ cells (green; proliferating cells) was quantified in the limbal, peripheral and central areas of naïve wild-type and *Has2^Δ^^/^^Δ^^CorEpi^* mice. *Has2^Δ^^/^^Δ^^CorEpi^* mice present increased cell proliferation in all regions of the cornea when compared with wild-type mice. The number of proliferating cells was also counted at the wound edge of the central circle wound 10 hours after the circle or circle and ring wound of wild-type (D) and *Has2^Δ^^/^^Δ^^CorEpi^* (E) mice. *Has2^Δ^^/^^Δ^^CorEpi^* mice presented a decrease in the number of proliferating cells at the wound edge after the circle and ring wound. The number of EdU^+^ and DAPI^+^ cells was quantified in the different zones of the corneas of wild-type (F, H, and I) and *Has2^Δ^^/^^Δ^^CorEpi^* (G, J, and K) mice after the central and ring wounds (F and G), 0.75 mm (H and J) and 1.5 mm (I and K) central wounds with and without concomitant removal of the limbal rim. The number of proliferating cells throughout the different corneal zones presented as a percentage of total cells. *P ≤ 0.05 and **P ≤ 0.01.

Cell proliferation was first analyzed in the different corneal zones of both naïve wild-type and *Has2^Δ^^/^^Δ^^CorEpi^* mice (Figs. 3A, B, and C and Supplemental Figs. 2 and 3). Naïve *Has2^Δ^^/^^Δ^^CorEpi^* mice presented higher proliferation rates in all of the corneal zones compared with naïve wild-type mice; however, this finding was more significant in the central and peripheral cornea (Figs. 3B and C). The number of proliferating cells was counted in the central cornea, peripheral cornea, and limbal region of both naïve wild-type and *Has2^Δ^^/^^Δ^^CorEpi^* mice. The highest number of EdU^+^ cells were identified in the peripheral cornea, with lower numbers in the central cornea and limbal region (Fig. 3B). Thus, the peripheral cornea clearly contributes toward the maintenance of uninjured corneas. Similar results were obtained when analyzing the ratio of EdU^+^ cells per total number of DAPI^+^ cells (Fig. 3C). The number of proliferating cells was also counted within 50 µm of the wound edge of the central circular wounds to verify whether the proliferation of central epithelial cells at the wound edge contributes toward the resurfacing of central wounds with and without a surrounding ring wound. Interestingly, for wild-type mice, the ring wound did not affect the number of proliferating cells at the wound edge when compared with the circle wound only (Fig. 3D). In contrast, *Has2^Δ^^/^^Δ^^CorEpi^* mice subjected to both circle and ring wounds presented a significant decrease in the number of proliferating cells at the wound edge when compared with those subjected to the central wound only (Fig. 3E). The percentage of proliferating cells was also calculated throughout the different corneal zones (Figs. 3F and G). Interestingly, in wild-type mice there is an increase in proliferating cells within the central and peripheral cornea (zones 3–4) in corneas subjected to the central and ring wounds, when compared with the central wounds alone (Fig. 3F). Thus, the ring wounds trigger an increase in proliferation in the central and inner peripheral cornea. In stark contrast, the ring wounds lead to a decrease in proliferation within the limbal region when compared with the central wounds alone (zones 7–8; Fig. 3F). It is important to note that the ring wound was made in zone 4. Differently to wild-type mice, *Has2^Δ^^/^^Δ^^CorEpi^* mice presented a decrease in proliferation throughout all zones when subjected to the central and ring wound when compared with the central wound alone (Fig. 3G).

### Circular Wounds Do Not Heal in a Homogeneous Manner

During this study, it could be noted that corneal wounds do not heal in a homogeneous manner. More specifically, it could be noted that epithelial cells move into the wounded area around the circumference of central corneal wounds at different rates. Thus, the upper, lower, nasal, and temporal regions of corneas were demarcated to evaluate which regions healed at a faster rate. Interestingly, we found that there was a tendency for the wound to close at a faster rate along the superior–inferior axis when compared with the nasal-temporal axis of the wound (Supplemental Fig. 4). Therefore, when studying the rate of corneal wound healing, investigators should ascertain the same regions of the cornea are analyzed in each sample to decrease intersample variability.

### Wound Size Dictates the Need for LSCs to Heal Corneal Wounds

**Figure 4.**
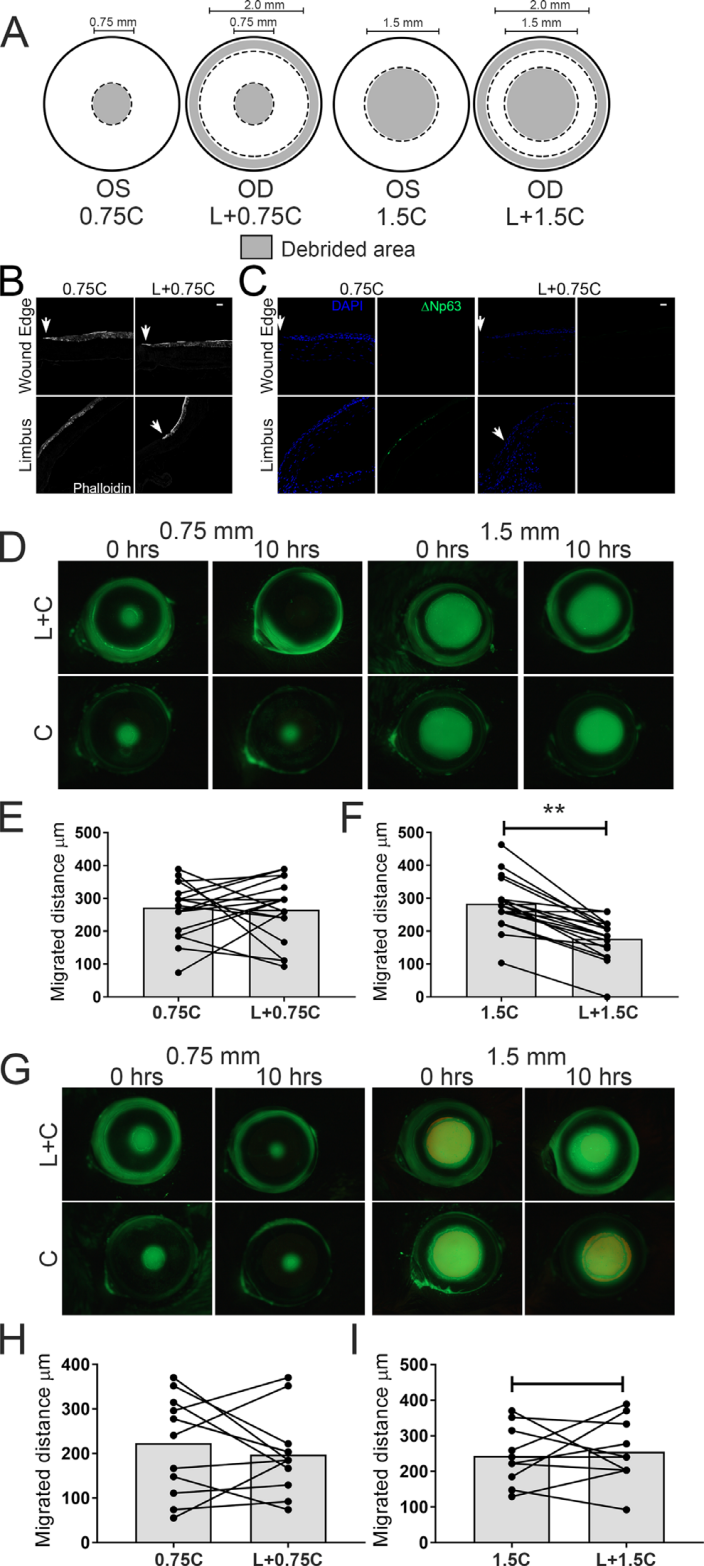
Participation of LSCs in healing small and large central corneal wounds in wild-type and *Has2^Δ^^/^^Δ^^CorEpi^* mice. (A) Schematic of the wounding model; the left eye was subjected to a central circular wound of 0.75 or 1.50 mm in diameter (0.75C or 1.5C, respectively), whereas the right eye was subjected to the same central wound, as well as removal of the limbal rim (0.75C+L or 1.5C+L, respectively). The debrided area is represented in gray and spared epithelium in white. Eyeballs were obtained 10 hours after injury, processed for histologic analysis and stained with phalloidin (B) and ΔNp63 (C). (D) Images were acquired of the ocular surface of wild-type mice treated with fluorescein under a stereomicroscope using the GFP filter immediately and 10 hours after injury. The migrated distance was calculated between 0 and 10 hours after injury for the 0.75 (E) and 1.50 mm wounds (F). Removal of the limbal area only affected the wound healing of larger central wounds, while the wound healing of smaller wounds was not affected. (G) Images were acquired of the ocular surface of *Has2^Δ^^/^^Δ^^CorEpi^* mice treated with fluorescein under a stereomicroscope using the GFP filter immediately and 10 hours after injury. The migrated distance was calculated immediately and 10 hours after injury for the 0.75 (H) and 1.50 mm wounds (I). Removal of the limbal area had no effect on the wound healing of smaller or larger central wounds in *Has2^Δ^^/^^Δ^^CorEpi^* mice. P^*^ ≤ 0.05 and ^**^P ≤ 0.01. Scale bar represents 20 µm.

**Figure 5.**
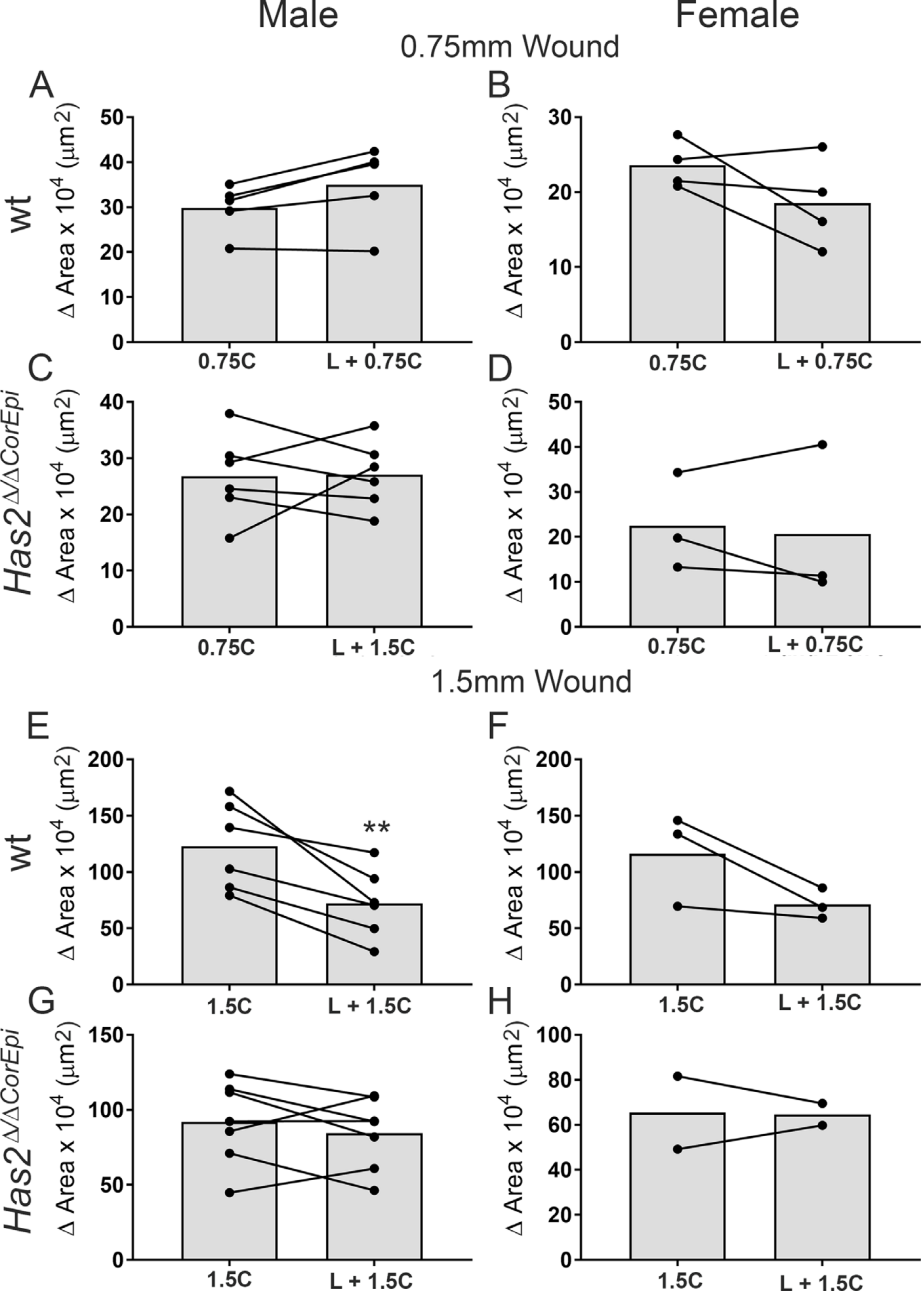
The participation of LSCs in corneal wound healing of differently sized central corneal wounds in male and female wild-type and *Has2^Δ^^/^^Δ^^CorEpi^* mice. The healed area for small and large central wounds was analyzed for wild-type and *Has2^Δ^^/^^Δ^^CorEpi^* mice with gender as a variable. (A) Male wild-type mice subjected to 0.75 mm central wounds, (B) female wild-type mice subjected to 0.75 mm central wounds, (C) male *Has2^Δ^^/^^Δ^^CorEpi^* mice subjected to 0.75 mm central wounds, (D) female *Has2^Δ^^/^^Δ^^CorEpi^* mice subjected to 0.75 mm central wounds, (E) male wild-type mice subjected to 1.50 mm central wounds, (F) female wild-type mice subjected to 1.50 mm central wounds, (G) male *Has2^Δ^^/^^Δ^^CorEpi^* mice subjected to 1.50 mm central wounds, and (H) female *Has2^Δ^^/^^Δ^^CorEpi^* mice subjected to 1.50 mm central wounds. *P ≤ 0.05 and **P ≤ 0.01.

Previous studies have come to divergent conclusions on whether LSCs are required to heal corneal wounds. Some studies have shown that LSCs are not necessary for maintenance of the central corneal epithelium^13^^,^^25^ and closure of mild corneal wounds,^8^ whereas other studies have shown that the loss of LSCs leads to severely impaired corneal wound healing.^26^^–^^29^ We hereby investigated whether wound size could play a role in dictating the need for LSCs in corneal wound closure. For such, the closure of small wounds (0.75 mm in diameter) and large wounds (1.50 mm in diameter) was compared with (right eye) and without (left eye) the removal of cells within the limbal rim (Fig. 4A), as previously shown.^30^^–^^34^ The successful removal of LSCs using our surgical procedure for debriding the limbal rim was confirmed immediately after wounding (results not shown) and also 10 hours after injury (Figs. 4B and C). The lack of LSCs was still evident at 10 hours after wounding (Figs. 4B and C). The wounded area clearly encompasses the limbal region (Fig. 4B) and ΔNp63^+^ cells can only be seen in the limbal epithelium of wild-type mice subjected to the central wound, although they cannot be seen after the removal of the limbal rim (Fig. 4C). Wild-type mice subjected to the small wound displayed similar rates of wound healing with or without the removal of epithelial cells within the corneal limbal rim, indicating that LSCs are not necessary for the closure of smaller wounds (Figs. 4D and E). In contrast, wild-type mice subjected to the larger injury displayed slower rates of wound healing when epithelial cells within the corneal limbal rim were removed, indicating that LSCs are necessary for closure of larger wounds (Figs. 4D and F). Interestingly, *Has2^Δ^^/^^Δ^^CorEpi^* mice presented similar wound healing with and without the removal of epithelial cells within the limbal region, both for smaller and larger wounds (Figs. 4G, H, and I). Thus, the removal of epithelial cells within the corneal limbal rim of *Has2^Δ^^/^^Δ^^CorEpi^* mice does not affect the rate of wound healing, irrespective of wound size. Interestingly, the size of the injury did not affect the distance migrated by epithelial cells. Specifically, in wild-type mice the migrated distance after the 0.75 mm central wound was approximately 275 µm and after the 1.5 mm injury approximately 280 µm (Figs. 4 E and F). In *Has2^Δ^^/^^Δ^^CorEpi^* mice, the migrated distance after the 0.75 mm central wound was approximately 220 µm and after the 1.5 mm injury approximately 240 µm (Figs. 4H and I). Therefore, the epithelial cells move at a similar rate to resurface corneal epithelial injuries irrespective of wound size. Gender was also analyzed as a variable in this experiment and differences were noted (Fig. 5 and Table 1). There was a tendency for male wild-type and *Has2^Δ^^/^^Δ^^CorEpi^* mice to display an increase in the wound healing rate for 0.75 mm injuries with the removal of cells within the limbal rim, whereas females displayed a decreased rate, although none of these differences achieved significance (Fig. 5 and Table 1). For the larger wounds, both male and female mice displayed similar healing patterns (Fig. 5 and Table 1). Importantly, the overall wound healing rate was significantly higher in male wild-type and *Has2^Δ^^/^^Δ^^CorEpi^* mice when compared with female mice, and these data achieved statistical significance (Fig. 5 and Table 1). Moreover, when comparing wound healing of the central wound of wild-type and *Has2^Δ^^/^^Δ^^CorEpi^* mice for small wounds, the average healed area was approximately 30 µm^2^ for both wild-type and *Has2^Δ^^/^^Δ^^CorEpi^* male mice and approximately 22 µm^2^ for both wild-type and *Has2^Δ^^/^^Δ^^CorEpi^* female mice. In contrast, for larger wounds, the average healed area was approximately 120 µm^2^ for wild-type male mice compared with approximately 90 µm^2^ for *Has2^Δ^^/^^Δ^^CorEpi^* male mice, and approximately 120 µm^2^ for female wild-type mice compared with approximately 65 µm^2^ for female *Has2^Δ^^/^^Δ^^CorEpi^* mice. Thus, *Has2^Δ^^/^^Δ^^CorEpi^* mice only present reduced wound healing compared with wild-type mice for larger wounds. This finding is consistent with our data showing that LSCs are only necessary for healing larger wounds; thus, mice that present a loss of LSCs only display delayed wound healing for larger wounds.

**Table 1.** Healed Area in Male and Female Wild-Type and Has2^Δ/ΔCorEpi mice^ After Small and Large Central Wounds Combined or Not Combined With Limbal Wounds

Wound type		Male	Female	*t* Test
Wt	0.75C	298028 *(54571)*	235793 *(31274)*	0.08
	L+0.75C	349678* *(90153)*	210477* *(41492)*	0.02
*Has2^Δ^^/^^Δ^^CorEpi^*	0.75C	251887 *(81241)*	224634 *(107565)*	0.66
	L+0.75C	270471 *(59550)*	206424 *(172201)*	0.41
wt	1.5C	1230202 *(389149)*	1164315 *(410551)*	0.82
	L+1.5C	722806 *(311680)*	711706 *(135888)*	0.95
*Has2^Δ^^/^^Δ^^CorEpi^*	1.5C	918332 *(302726)*	841993 *(268636)*	0.69
	L+1.5C	845578 *(236279)*	565842 *(148683)*	0.09

Healed area is presented as µm^2^ (SD). Statistically significant values are indicated with an *. Statistical analysis was carried out between the male and female mice for each injury type.

### The Effect of Wound Size on Corneal Epithelial Proliferation When LSCs Are Spared or Not Spared

The number of proliferating cells was quantified within concentric circular rings placed at 0.25 mm intervals throughout the cornea (Figs. 3H–K). The number of EdU^+^ and DAPI^+^ cells was calculated in the different zones of the cornea and data presented as the percentage of EdU^+^ cells in relation to the total number of cells (i.e., DAPI^+^ cells). There was an overall decrease in the number of proliferating cells in wild-type corneas subjected to the larger central wound (1.5 mm) and/or limbal wounds when compared with naïve wild-type corneas (Figs. 3H and I). When wild-type mice were subjected to the 0.75 mm central wound alone, there was an increase in proliferation within the central cornea (zones 1–4) when compared with naïve mice. Thus, larger corneal injuries or injuries to the limbal region lead to an overall decrease in proliferating cells throughout all zones of wild-type corneas (Figs. 4H and I). Moreover, combined central and limbal wounds lead to a more pronounced reduction in proliferation throughout all corneal zones when compared with solely the central wound. Interestingly, it can be noted that when mice are subjected to the smaller central wound (0.75 mm) with and without the removal of the limbal rim, the profile of proliferating cells throughout the cornea is very different to that of mice subjected to the larger wounds (1.5 mm). Mice subjected to the central 0.75 mm wounds, with or without the limbal wounds, present a similar number of proliferating cells throughout all zones, although these values are significantly lower in the corneas that were also subjected to limbal wounds. It is important to note that the limbal rim (which was removed during limbal injury) falls within zones 7 and 8. For *Has2^Δ^^/^^Δ^^CorEpi^* mice, there was an increase in proliferation within the central and peripheral cornea (specifically zones 3–6) after the 0.75 mm wounds with or without the removal of the limbal rim. After the circular 1.5 mm wound alone, there was an increase in proliferation within zones 4 to 7; however, for circular 1.5 mm wounds combined with the removal of the limbal rim there was a decrease in proliferation in zones 2, 3, 6, and 7 when compared with naïve mice (Figs. 3J and K). Thus, with smaller wounds, removal of the limbal rim had no effect on cell proliferation in the peripheral cornea; however, when combining the larger central wound with the limbal wound, there was a decrease in proliferation within the peripheral cornea, similar to what was observed in wild-type mice.

### The Effect of Injury Type and Size on Corneal Stratification

**Figure 6.**
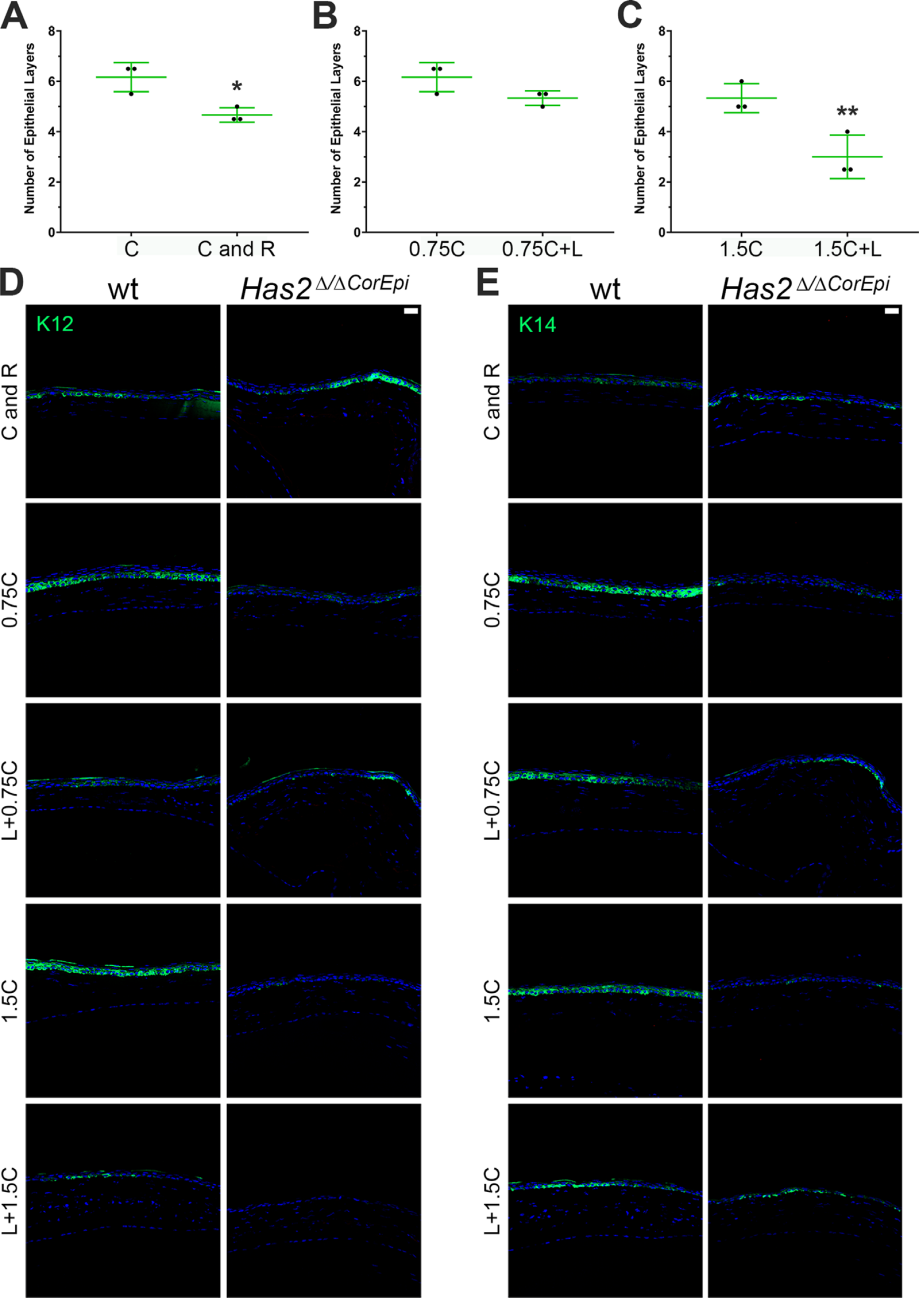
The effect of different corneal wounds on corneal stratification. Wild-type mice were subjected to different corneal wounds and culled after 48 hours. The eyeballs were processed for histologic analysis and the number of epithelial layers counted in the central cornea. The number of epithelial layers were counted in wild-type mice subjected to circular or circular and ring wounds (A), wild-type mice subjected to 0.75 mm central circular wounds with or without the removal of the limbal rim (B), and wild-type mice subjected to 1.5 mm central circular wounds with or without the removal of the limbal rim (C). Histologic sections were also processed for immunohistochemistry and stained with K12 (D) and K14 (E). ^*^P ≤ 0.05 and ^**^P ≤ 0.01. Scale bar = 20 µm.

To verify the effect of the different wounds on corneal stratification, corneas were obtained 48 hours after injury and processed for histologic analysis. The histologic sections were stained with hematoxylin and eosin, the central cornea was imaged under a light microscope, and the number of epithelial cell layers was counted in a blinded manner by two independent investigators. In wild-type mice, the ring wound surrounding the corneal central wound led to a reduction in the number of cell layers 48 hours after injury, specifically the mice subjected to solely the central wound presented approximately six layers while the corneas subjected to both the central and ring wound presented approximately five layers (Fig. 6A). This finding is consistent with the fact that the ring wound also delayed the closure of the central corneal wound (Fig. 1). The removal of the limbal rim did not affect the corneal stratification after the 0.75 mm central wound; however, the removal of the limbal rim significantly decreased the number of cell layers after the 1.5 mm wound from approximately 5.5 layers to 3.0 layers (Figs. 6B and C). This finding is also consistent with the fact that the removal of the limbal rim only affected the rate of wound healing for the larger (1.5 mm) wounds (Fig. 4). We also analyzed the distribution of K12- and K14-positive cells within the central cornea of wild-type and *Has2^Δ^^/^^Δ^^CorEpi^* mice 48 hours after injury (Figs. 6D and E, respectively). In wild-type mice, K12 and K14 staining show the formation of a fully stratified epithelium 48 hours after 0.75 and 1.50 mm central wounds. However, both the ring wound and the removal of epithelial cells within the limbal rim delay the formation of a fully stratified epithelium in wild-type mice (Figs. 6D and E). Interestingly, the distribution of K12- and K14-positive cells within the central cornea of *Has2^Δ^^/^^Δ^^CorEpi^* mice subjected to 0.75 and 1.50 mm wounds resembles that of wild-type mice subjected to 0.75 and 1.50 mm wounds in conjunction with removal of the limbal rim. Thus, these data support the finding that the wound healing process in *Has2^Δ^^/^^Δ^^CorEpi^* mice resembles that of wild-type mice after the removal of epithelial cells within the limbal rim.

## Discussion

This study used different types of epithelial debridement wounds to assess the contribution of limbal, peripheral, and central corneal epithelial cells in repairing central epithelial injuries. Over the years, various divergent wound healing models have been proposed to explain the process by which epithelial cells resurface the cornea after injury. For example, it has been proposed that LSCs constantly replenish the corneal epithelium to maintain homeostasis^35^^–^^37^ and increased proliferation from the limbus drives the centripetal movement of corneal epithelial cells during wound healing.^14^^,^^19^ In the absence of the limbal epithelium, transient-amplifying cells or corneal progenitor cells are believed to have limited proliferative capacity to maintain homeostasis for short periods.^8^^,^^38^^,^^39^ However, other studies have shown that the basal layer of the entire murine corneal epithelium consists of oligopotent stem cells expressing putative stem cell markers^7^^,^^13^ and that corneal-committed cells can move centrifugally, dedifferentiate, and even repopulate the LSC pool.^9^^,^^40^^,^^41^ It is important to note that, although some centrifugal movement has been shown to exist during the process of wound healing, it has not been shown to exist during homeostasis. One aim of this study was to understand some of the technical differences that could have led to distinct findings by different groups.

Our data comparing central corneal wounds to central corneal wounds surrounded by a peripheral ring wound, which prevents the migration of LSCs into the central cornea and also prevents cells from being pushed from the limbal region into the central cornea, clearly show that epithelial cells contribute toward closure of central wounds. Our data show that the ring wound delays wound healing in wild-type mice; however, it does not prevent wound healing, indicating that, although LSCs are important for wound closure, peripheral epithelial cells also contribute toward wound healing. Thus, the cornea does not rely exclusively on LSCs for resurfacing corneal injuries. Previous studies using a mathematical simulation model along with in vivo observations have shown that a population pressure gradient from the limbus causes the centripetal movement of cells toward the central cornea.^19^^,^^42^ Our findings suggest this is not the only source of cells during the initial phases of corneal epithelial wound healing. Specifically, our proliferation data show that, during the initial phase of wound healing (6–10 hours), there was no significant increase in proliferation within the limbal region, for both small and large central circular injuries. Thus, the theory suggesting that a population pressure gradient within the limbal region is responsible for pushing the basal cells to resurface the corneal epithelium after a debridement wound during the initial phase of wound healing is not supported by our data. Interestingly, our *Has2^Δ^^/^^Δ^^CorEpi^* mice did not present a significant difference in wound healing of the central corneal wound with or without the ring wound. Thus, impeding the migration of cells within the limbal region onto the corneal epithelium had a limited effect on wound healing in these mice. This finding indicates that these mice, which present a loss of LSCs, rely heavily on peripheral epithelial cells for wound closure. Our data also show that naïve *Has2^Δ^^/^^Δ^^CorEpi^* mice present higher levels of cell proliferation in all regions of the cornea, namely, the limbal, peripheral, and central regions. Therefore, our data suggest that because these mice have deficient LSCs, they also rely on corneal epithelial cells for maintenance of the cornea. Our previous work showed that naïve *Has2^Δ^^CorEpi^* mice present a similar number of epithelial layers as wild-type mice; thus, the significantly increased cell proliferation throughout these corneas indicates they have an increased rate of epithelial cells sloughing off.^20^ Thus, our new data indicate that corneal epithelial cells alone are not as efficient at maintaining the corneal epithelium as LSCs.

Our injury models comparing the wound closure of central wounds of different sizes, with and without concomitant removal of LSCs, clearly demonstrate that the wounded area of central corneal wounds dictates whether LSCs are necessary for healing a corneal wound in wild-type mice. Specifically, smaller wounds do not trigger the migration of cells from the limbal region into the central cornea and, instead, peripheral and central epithelial cells resurface the injured area. In contrast, larger central corneal wounds trigger the migration of cells from the limbal region into the central cornea. These data can explain why different groups have reported divergent findings regarding the need for LSCs to heal central wounds (summarized in Table 2). Interestingly, *Has2^Δ^^/^^Δ^^CorEpi^* mice did not present a difference in wound healing with or without removal of the limbal region, irrespective of wound size. Therefore, in this experimental mouse model of LSCD, removal of the limbal region does not affect wound healing for either small or large central wounds. Moreover, our data from central and ring wounds indicate that the peripheral cornea is the major contributor toward wound healing in these mice, and thus, *Has2^Δ^^/^^Δ^^CorEpi^* mice rely mostly on corneal epithelial cells to maintain the cornea during homeostasis and for healing the cornea after injuries. This study also supports our previous findings suggesting *Has2^Δ^^CorEpi^* mice are a good mouse model for LSCD.^20^

**Table 2.** Previous Studies Analyzing the Dynamics of Corneal Wound Healing

Reference	Species	Wound Details
Hou et al., 2020 ^43^	C57BL/6J mice	2.5-mm diameter scraped using a blade
Okada et al., 2020 ^44^	Compound transgenic mice	2-mm diameter using microsurgical blade
Murataeva et al., 2019 ^45^	C57BL/6J and CD1 mice	∼1-mm diameter using an Alger Brush
Park et al. 2019 ^19^	K14CreER-Confetti mice	2-mm diameter using Algerbrush with 1-mm burr
Nasser et al., 2018 ^9^	K15-GFP/Confetti mice	Limbal removal with Algerbrush
Chen et al., 2017 ^46^	Male C57BL/6 mice	3-mm diameter using Algerbrush II
Zhang et al., 2017 ^47^	Female C57BL/6 mice	2-mm diameter trephine and golf club spud
Huang et al., 2017 ^48^	Sprague-Dawley rats	4-mm diameter using Algerbrush II with 0.5-mm burr
Walczysko et al., 2016 ^49^	Transgenic mice on the CBA/Ca genetic background	1-mm diameter scraped using scalpel blade
Rush et al., 2016 ^50^	Female C57BL6/J mice	1.5-mm diameter using Algerbrush II with 0.5-mm burr
Amitai-Lange et al., 2015 ^8^	R26R-Confetti mice	DMSO injury
Martin et al., 2013 ^51^	Wistar male adult rats	1-mm diameter cauterization with silver nitrate sticks
Kawakita et al. 2011 ^39^	Japanese white rabbit	4-, 5-, 6-, and 8-mm diameter
Yang et al., 2010 ^52^	Female NZ white rabbits	6-mm diameter using No. 15 scalpel blade
de Faria-e-Sousa et al., 2010 ^40^	NZ white rabbit	Epithelium scraped outside central 6- mm diameter with ophthalmic spatula
Majo et al., 2008 ^13^	Wild-type, OF1, Athymic and SCID mice	1.5 mm × 0.3 mm of the limbus of athymic mice were excised and transplanted with 1.5-mm diameter wound using spatula
Li et al., 2004 ^53^	Male C57BL/6 mice	2-mm diameter trephine and diamond blade for refractive surgery (Accutome)
Danjo and Gipson 2002 ^17^	Male Balb/c mice 6-9 weeks	2.0- or 2.5-mm diameter corneal epithelium debrided with a blunted blade, leaving the basement membrane intact
Huang and Tseng, 1991 ^38^	NZ white rabbit	7.5-mm trephine and surgical blade used twice
Crosson et al., 1986 ^54^	NZ white rabbit	2-, 4-, or 6-mm diameter
Buck RC. 1979 ^14^	Male or female Swiss mice	Small circular 1.0- to 1.4-mm diameter or large ∼3-mm diameter irregular or elongated wounds of denuded epithelium by repeatedly pressing against the proptosed eye using a microscope slide previously coated with 10% gelatin solution and allowed to dry
Kuwabara et al., 1976 ^16^	NZ albino rabbits	Linear stromal wound 5 mm long and 0.2 mm deep
Hanna C. 1966 ^15^	Adult albino rabbits and rats	1 mm wide and 5 mm long, 1.5 mm from limbus

Various previous studies have worked toward unveiling the mechanism by which stem cells maintain the cornea during homeostasis and heal the cornea after injury. The most widely accepted model is the XYZ hypothesis, which states that terminally differentiated cells slough of the ocular surface (Z), and these are replenished by the proliferation (X) and centripetal migration (Y) of stem cells in the limbal basal epithelium.^3^^,^^6^^,^^18^^,^^29^^,^^39^^,^^42^ Although these studies indicate LSCs are located exclusively in the limbal region of mice, Majo et al.^13^ have shown that stem cells are not limited to the limbal region, and instead clusters of stem cells exist within the cornea. Although numerous studies have disputed these findings, others have suggested there could be a leakage of stem cells from the limbus into the central cornea, and therefore the central cornea could contain oligopotent cells that can be self-maintained and contribute to mild wound repair for several months.^13^^,^^19^^,^^42^ Whether it is these oligopotent cells or corneal central and peripheral epithelial cells themselves that contribute toward wound closure without LSC participation in our model remains to be established. Importantly, we believe our findings shed light on why independent groups have come to differing conclusions about the involvement of LSCs during homeostasis and wound healing. Specifically, our data show that the size and exact location of the wound on the corneal epithelium directly impacts the directionality of epithelial cell migration and involvement of LSCs in the wound healing process, as well as whether there is an increase or decrease in proliferation throughout the different zones of the cornea during the early stages of wound healing.

Previous studies have investigated the direction of epithelial cell movement, including tracing the tracks of LSCs as they move into the cornea. The presence of slow cycling cells at the basal limbal epithelium that move centripetally toward the central cornea under normal physiology and after injury has been previously shown using DNA labeling.^6^ The centripetal movement of these cells has also been studied using India ink dye^55^ and mice expressing GFP ubiquitously driven by a CAG^37^ or β-actin promoter.^35^ More recently, lineage tracing experiments driven by epithelium-specific promoters like keratin 5,^56^ keratin 12,^24^^,^^57^ keratin 14,^8^^,^^19^^,^^36^ and keratin 15^9^ showed that radial streaks arise from the limbus and proceed toward the central cornea. Although general consensus leans toward the hypothesis that epithelial cells move only in the centripetal direction, some studies have shown that centrifugal cell movement exists during corneal wound healing of mouse,^9^ rabbit,^40^ and human^41^ corneas. The central epithelium of rabbit corneas was capable of regenerating by centrifugal movement after seven consecutive debridements of the peripheral epithelium outside the central 6 mm diameter circle.^40^ A human corneal organotypic culture model has shown that epithelial cells could regrow in both the periphery inward and center outward direction to the limbus.^41^ The murine corneal epithelium has not only demonstrated the ability to move centrifugally to the denuded limbal region but also to repopulate the stem cell population in the presence of intact stroma.^9^ Our study revealed that both centripetal and centrifugal movement of epithelial cells is possible in the cornea during wound healing. Our data show that ring wounds heal in both the limbus to central cornea direction and the central cornea to limbus direction. Thus, if a cornea injury exists in the periphery of the cornea, epithelial cells in the central cornea can move in a centrifugal fashion to resurface the wound. Recently, Park et al.^24^ used a lineage tracing model to demonstrate that ring injuries heal primarily from the limbal region via centripetal movement of epithelial cells, with significantly lower centrifugal movement of central corneal epithelial cells into the wound bed. They also noted that during the later phase of wound healing, specifically 16 to 20 hours after wounding, proliferation was significantly increased only in the limbal region and not in the central cornea.^24^ There are important differences between this work and our current study, specifically we studied an early phase of corneal wound healing in vivo, whereas Park et al. studied a late phase of wound healing using ex vivo, in vivo, and computational corneal wound healing models. Importantly, previous studies have shown that proliferation within the peripheral cornea and limbal region is up-regulated during later phases of wound healing and is important primarily during the stratification process.^19^^,^^54^

Finally, when comparing the dynamics of wound closure in live mice it was noted that the circular shape of the injury was lost over time as injuries heal, indicating that the rate of wound healing is not even throughout the circumference of the wound. We found that the wound generally heals at a faster rate in the superior–inferior axis region. This pattern could possibly be due to eyelids covering these areas and/or the sweeping motion of eyelids over epithelial cells while blinking. A similar pattern of epithelial wound healing was also observed in other studies using mice,^58^ rats,^59^ and rabbits.^60^ The Borderie group has shown that, in humans, the limbal crypts containing LSCs are more preponderant in the vertical axis of the cornea than in the horizontal axis.^61^ Interestingly, Shortt et al.^62^ demonstrated that LSC niche structures are located only in the superior and inferior limbal quadrants, being absent in the nasal and temporal quadrants. The nasal and temporal quadrants are the areas in the horizontal meridian of the eye that are most exposed to sunlight, which could be why they lack LSCs.^62^ Thus, the differential distribution of LSC clusters throughout the corneal perimeter could be responsible for the faster rate of wound healing in the superior–inferior axis. It is important to note that there are significant differences in the architecture of the limbal region and in LSC composition between human and mouse corneas.^7^^,^^13^^,^^61^^,^^63^ However, studies using animal models provide invaluable information on the biology of LSCs and their role in wound healing.

Taken together, this study clearly demonstrates that the shape and dimensions of a corneal epithelial injury dictate the mechanism by which corneas are healed. Both LSCs and epithelial cells migrate toward the wounded area. Thus, epithelial cells have the ability to move toward the injured area in both a centripetal and/or centrifugal manner, depending on where the injury is located in reference to the center of the cornea. Importantly, our work reveals that corneas with deficient LSCs rely heavily on corneal epithelial cells to resurface a corneal wound, and that these peripheral epithelial cells are capable of resurfacing the corneal epithelium of these mice. Finally, we clearly show that the dimensions of the corneal wound dictates the involvement of LSCs in the wound healing process.

## Supplementary Material

Supplement 1

Supplement 2

Supplement 3

Supplement 4
