# Supplementary material for: Epithelial Cell Migration and Proliferation Patterns During Initial Wound Closure in Normal Mice and an Experimental Model of Limbal Stem Cell Deficiency
Source: Invest Ophthalmol Vis Sci. 2020 Aug 13;61(10):27. doi: 10.1167/iovs.61.10.27 (PMC7441334; doi:10.1167/iovs.61.10.27)
Supplement: Supplement 1 [file iovs-61-10-27_s001.pdf]

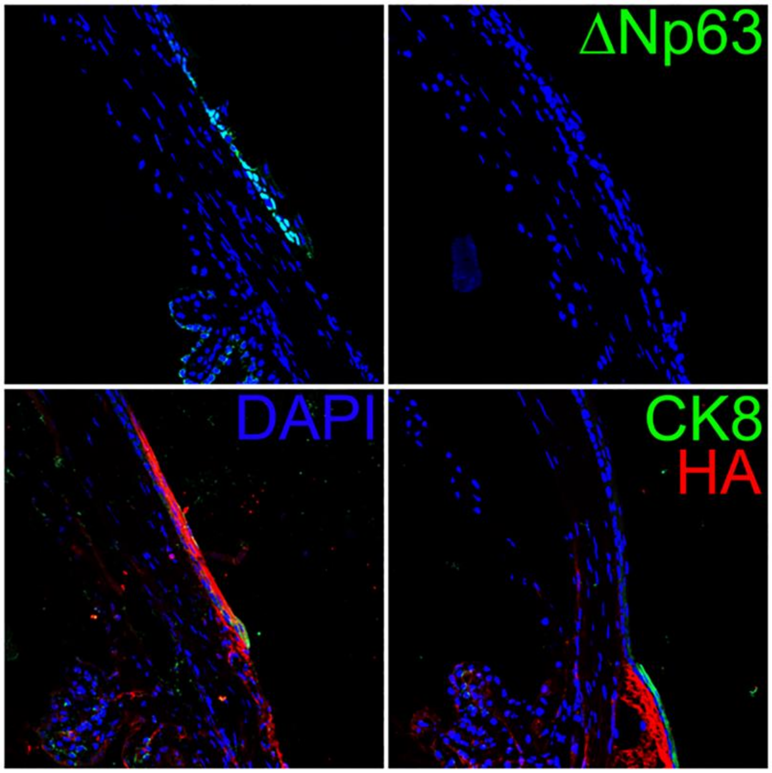

Supplemental Figure 1

Loss of LSCs in *Has2*$^{\Delta/\Delta CorEpi}$ mice. Corneas of wild-type and *Has2*$^{\Delta/\Delta CorEpi}$ mice were subjected to immunohistochemistry analysis and the limbal region imaged. LSCs were identified using anti-ΔNp63 (green - upper panels) and anti-CK8 (green - lower panels). HA was stained using biotinylated HABP. The nuclei were counterstained with DAPI (blue).
