# Supplementary material for: Epithelial Cell Migration and Proliferation Patterns During Initial Wound Closure in Normal Mice and an Experimental Model of Limbal Stem Cell Deficiency
Source: Invest Ophthalmol Vis Sci. 2020 Aug 13;61(10):27. doi: 10.1167/iovs.61.10.27 (PMC7441334; doi:10.1167/iovs.61.10.27)
Supplement: Supplement 2 [file iovs-61-10-27_s002.pdf]

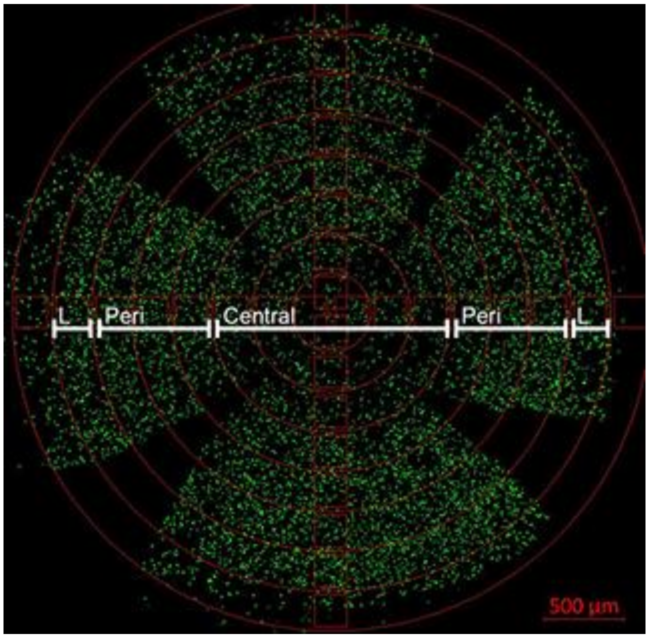

Supplemental Figure 2

Representative image of the demarcated zones used to quantify the number of EdU$^+$ cells. The corneal wholemounts with EdU labeling were overlaid with concentric circles placed at 0.25 mm intervals and the area inside inner three circles were considered as the central cornea (Central), the area inside next three circles as the peripheral cornea (Peri) and the next circle was considered the limbal region (L).
