# Supplementary material for: Epithelial Cell Migration and Proliferation Patterns During Initial Wound Closure in Normal Mice and an Experimental Model of Limbal Stem Cell Deficiency
Source: Invest Ophthalmol Vis Sci. 2020 Aug 13;61(10):27. doi: 10.1167/iovs.61.10.27 (PMC7441334; doi:10.1167/iovs.61.10.27)
Supplement: Supplement 3 [file iovs-61-10-27_s003.pdf]

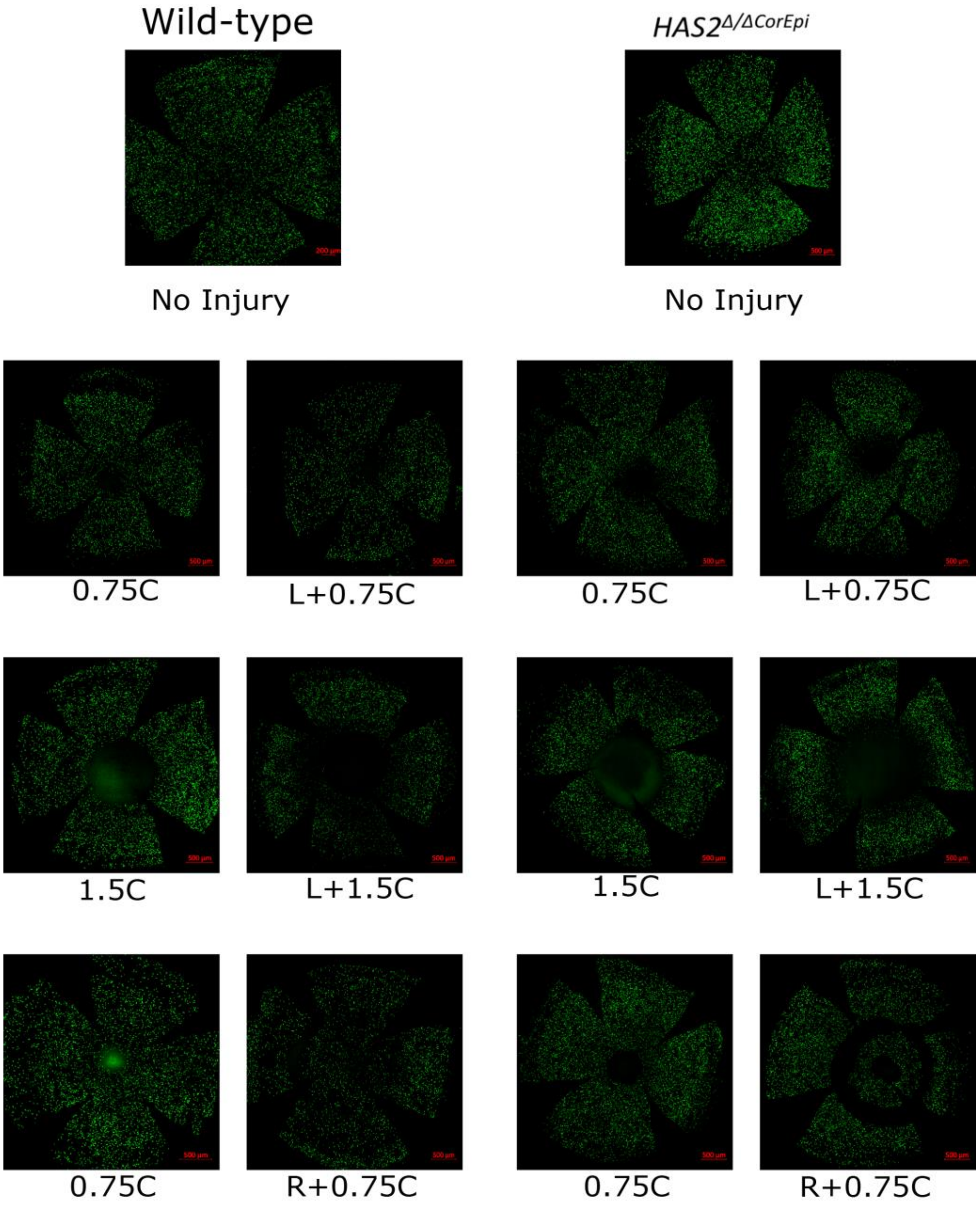


Supplemental Figure 3

**Representative images of the scanned whole mount EdU stained corneas for the different corneal injuries.** Wild-type and *Has2*$^{Δ/ΔCorEpi}$ mice were subjected to central 0.75 and 1.5 mm wounds with or without the removal of the limbal rim or ring wound.
