# Supplementary material for: Epithelial Cell Migration and Proliferation Patterns During Initial Wound Closure in Normal Mice and an Experimental Model of Limbal Stem Cell Deficiency
Source: Invest Ophthalmol Vis Sci. 2020 Aug 13;61(10):27. doi: 10.1167/iovs.61.10.27 (PMC7441334; doi:10.1167/iovs.61.10.27)
Supplement: Supplement 4 [file iovs-61-10-27_s004.pdf]

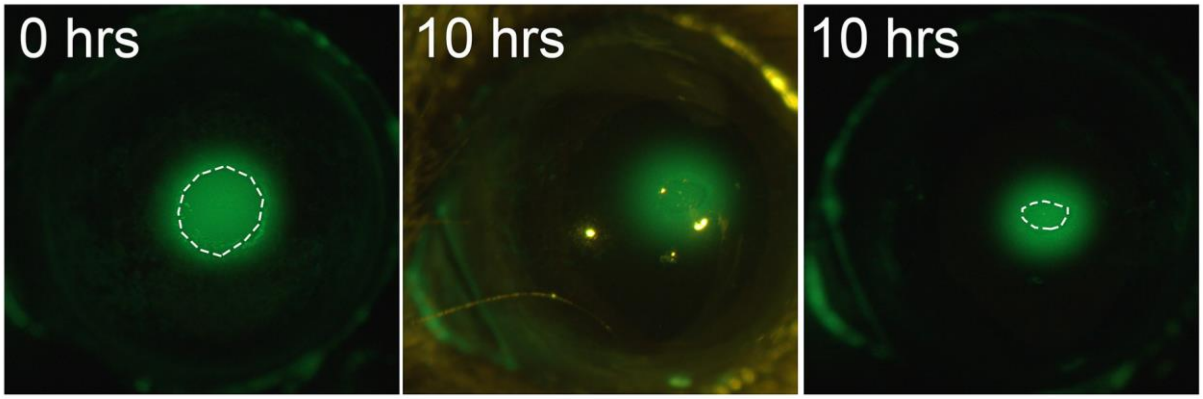

Supplemental Figure 4

Corneal injuries heal at a faster rate in the superior-inferior axis of the wound. The central corneal injured area of wild-type mice was observed over time revealing that corneal wounds heal at a faster rate in the superior-inferior axis when compared to the nasal-temporal axis of the wound.
